# Effect of a “Dual Trigger” Using a GnRH Agonist and hCG on the Cumulative Live-Birth Rate for Normal Responders in GnRH-Antagonist Cycles

**DOI:** 10.3389/fmed.2021.683210

**Published:** 2021-05-25

**Authors:** Fumei Gao, Yanbin Wang, Min Fu, Qiuxiang Zhang, Yumeng Ren, Huan Shen, Hongjing Han

**Affiliations:** Reproductive Center of Peking University Peoples' Hospital, Beijing, China

**Keywords:** dual trigger, GnRH antagonist protocols, normal responders, cumulative live birth rate, IVF

## Abstract

“Dual triggering” for final oocyte maturation using a combination of a gonadotropin-releasing hormone agonist (GnRHa) and human chorionic gonadotropin (hCG) can improve clinical outcomes in high responders during *in vitro* fertilization–intracytoplasmic sperm injection (IVF–ICSI) GnRH-antagonist cycles. However, whether this dual trigger is also beneficial to normal responders is not known. We retrospectively analyzed the data generated from 469 normal responders from 1 January to 31 December 2017. The final oocyte maturation was undertaken with a dual trigger with a GnRHa combined with hCG (*n* = 270) or hCG alone (*n* = 199). Patients were followed up for 3 years. The cumulative live-birth rate was calculated as the first live birth achieved after all cycles having an embryo transfer (cycles using fresh embryos and frozen–thawed embryos) among both groups. Women in the dual-trigger group achieved a slightly higher number of oocytes retrieved (11.24 vs. 10.24), higher number of two-pronuclear (2PN) embryos (8.37 vs. 7.67) and a higher number of embryos available (4.45 vs. 4.03). However, the cumulative live-birth rate and the all-inclusive success rate for assisted reproductive technology was similar between the two groups (54.07 vs. 59.30%). We showed that a dual trigger was not superior to a hCG-alone trigger for normal responders in GnRH-antagonist cycles in terms of the cumulative live-birth rate.

## Introduction

In cycles involving gonadotropin-releasing hormone antagonists (GnRHas), human chorionic gonadotropin (hCG) is used routinely to induce final oocyte maturation (1). However, hCG administration results in supraphysiologic steroid levels in the luteal phase due to its long half-life. Thus, hCG administration is associated with an increased risk of ovarian hyperstimulation syndrome (OHSS) (2). In the 1990s, to eliminate the risk of OHSS, GnRHas were introduced to promote final oocyte maturation in GnRH antagonist cycles (3). A single bolus of GnRHa can stimulate the release of luteinizing hormone (LH) and follicle-stimulating hormone (FSH) from the pituitary gland (4) to mimic the natural mid-cycle LH surge required for final oocyte maturation. Compared with administration of exogenous hCG, a GnRHa-induced LH surge has a shorter duration and smaller amplitude, which may help to reduce the risk of OHSS (3, 5). However, a significantly reduced implantation rate and higher abortion rate have been observed for cycles triggered by a lone GnRHa due to defective luteal phase function and decreased endometrial receptivity (6, 7). Therefore, a “dual trigger” method combining a single dose of a GnRHa with a reduced dose of hCG has been proposed to minimize the risk of OHSS and to improve clinical outcomes (8).

Several studies focusing on high responders have demonstrated significant improvements in ongoing pregnancy rates (9) and live-birth rates (10) when a dual trigger was used instead of a lone trigger of a GnRHa, all without conferring a significant increase in the OHSS rate. However, there is insufficient evidence regarding the impact of a dual trigger on the reproductive outcome in normal responders. Some studies reported a significantly improved ongoing pregnancy rate and live-birth rate in fresh-embryo transfer (FET) cycles for a dual-trigger group compared with the hCG-trigger group in normal responders (1, 11). By contrast, several studies have demonstrated that dual trigger of oocyte maturation was not associated with a change in the live-birth rate in FET cycles for normal ovarian responders (12, 13). However, those studies did not focus on the cumulative live-birth rate, which provides an all-inclusive success rate for assisted reproductive technology (ART).

We wished to investigate if dual triggering of final oocyte maturation with a combination of a single dose of a GnRH agonist and a low dose of hCG could improve the cumulative live-birth rate for normal responders in GnRH-antagonist *in vitro* fertilization–intracytoplasmic sperm injection (IVF–ICSI) cycles.

## Materials and Methods

### Ethical Approval of the Study Protocol

The study protocol was approved by the ethics review board of Peking University Peoples' Hospital (Beijing, China). All patients provided written informed consent. All treatments were undertaken in strict accordance with the Declaration of Helsinki 1964 and its later amendments.

### Study Design

In the present retrospective cohort study, a review of medical records from 1 January to 31 December 2017 was done for all IVF–ICSI cycles involving a GnRH-antagonist protocol at the Reproductive Center of Peking University Peoples' Hospital.

### Inclusion Criteria

The inclusion criteria were women: (i) aged <40 years; (ii) with a body mass index (BMI) of 18–30 kg/m^2^; (iii) who had a normal response to controlled ovarian stimulation (4–20 retrieved oocytes).

To evaluate the effect of a dual trigger on the cumulative live birth rate until the first live birth, only women who achieved the first live birth or had used up all of the fresh and frozen embryos acquired from a GnRH antagonist-stimulated cycle were included.

### Exclusion Criteria

The exclusion criteria were women: (i) with a strong response (number of retrieved oocytes >20) or weak response (number of retrieved oocytes <4) to controlled ovarian hyperstimulation (COH); (ii) with occult ovarian failure (FSH concentration ≥10 IU/L on day-3); (iii) with >3 attempts at IVF and/or ICSI; (iv) suffering from an endocrine disorder (diabetes mellitus, hyperprolactinemia, thyroid dysfunction, congenital adrenal hyperplasia, Cushing syndrome, or polycystic ovary syndrome), or a uterine anomaly confirmed by hysterosalpingography or hysteroscopy.

In this study, 1,021 patients who received the GnRH antagonist protocol for IVF/ICSI had the result of cumulative pregnancy outcome. Six hundred twenty-one patients who met the enrollment conditions were included. Seventy-eight patients with FSH ≥10 IU/L on day-3, 20 patients with >3 attempts at IVF and/or ICSI and 54 patients with endocrine disorders were excluded. Finally, 469 patients were included in the analysis.

### Protocols for Ovarian Stimulation

All patients received a flexible protocol involving the GnRH-antagonist for COH and they did not receive the oral contraceptive pill before the IVF cycle. Ovarian stimulation began on day-2 of the menstrual cycle with recombinant FSH (150–225 IU daily; Gonal-F; Merck Serono, Coinsins, Switzerland) for 3 consecutive days. The starting dose was determined by patient age, ovarian reserve, BMI, and previous response to COH. Then, the dose of recombinant FSH was adjusted according to the serum level of estrogen (E2) and follicular growth as monitored by serial transvaginal ultrasound. Administration of the GnRH-antagonist (0.25 mg of Ganirelix or Cetrotide given at 10 a.m. daily) was initiated based on a flexible protocol (in general when the lead follicle was 13–14 mm in diameter) and was continued until the day of hCG administration. When ≥2 leading follicles had reached 18 mm in diameter, final oocyte maturation was triggered by 250 mg of recombinant hCG (Ovidrel; Merck Serono) alone, which was equivalent to ~6,500 IU hCG according to manufacturer data, or by 0.2 mg of triptorelin (Ferring International Center, Saint-Prex, Switzerland) plus 2000 IU of hCG (Livzon, Zhuhai, China). Oocyte retrieval was undertaken by transvaginal ultrasonography 35–37-h later. ICSI was carried out for patients experiencing severe male-factor infertility.

### Embryo Culture

Fertilization (i.e., appearance of two distinct pronuclei and two polar bodies) was assessed 16–18 h after insemination. Zygotes were cultured in a cleavage medium (Cook Medical, Dublin, Ireland). Embryonic development was assessed daily. Transfer of fresh embryos was done 3 days after oocyte retrieval. Supernumerary embryos of excellent quality were cryopreserved (vitrification protocol). “Excellent quality” embryos were those that met three criteria: (i) 7–8 cells at 3 days after fertilization; (ii) <10% were fragmented; (iii) homogenous blastomeres.

If the criteria of excellent-quality embryos were not met, then embryos were cultured to the blastocyst stage in blastocyst medium (Cook Medical). Scoring for blastocyst quality was done on day-5 based on the Gardner classification (14). The score was dependent upon blastocyst expansion, development of the inner cell mass, and trophectoderm appearance. Scoring for the inner cell mass and trophectoderm was undertaken and, according to their morphologic appearance, blastocysts were graded as “top quality” (grade 1) (AA), “good quality” (grade 2) (AB and BA), “average quality” (grade 3) (AC, CA, BB), or “poor quality” (grade 4) (BC, CB, CC). Blastocysts with a score >3BB were vitrified on day-5 or−6. All embryos were frozen if a patient had issues related to a thin endometrial lining (<7 mm), intrauterine fluid, hydrosalpinx, increased progesterone level (>1.5 ng/mL) on the day of hCG administration, or had a high risk of ovarian hyperstimulation.

### Embryo Transfer

Transfer of fresh embryos was done 3 days after oocyte retrieval. In frozen–thawed embryo-transfer (FET) cycles, embryos were transferred in natural cycles or in hormonal-replacement cycles. Women who failed to ovulate were given estradiol valerate (Progynova; 3 mg, p.o., b.d.; Bayer, Leverkusen, Germany) from days 2 to 3 of the menstrual cycle. Progesterone (60 mg, i.m., once daily) was administered when the thickness of the endometrium reached ≥8 mm. The number of transferred embryos was 1–2 depending on embryo quality and patient age.

### Support During the Luteal Phase

For FET cycles, support during the luteal phase comprised daily intramuscular injection of 40 mg of progesterone in oil (Xianju, Taizhou, China) along with oral supplementation with 30 mg of dydrogesterone (Duphaston, Abbot Biologicals, Olst, the Netherlands), starting on the day of oocyte retrieval. For FTET cycles, intramuscular injection of 60 mg of progesterone in oil (Xianju) was undertaken from the day of endometrial transformation. The serum level of β-hCG was measured 14 days after embryo transfer, and a value >5 IU/mL was considered to denote clinical pregnancy. Luteal support was continued until 10 weeks of pregnancy.

### Outcome Variables

The primary outcome measure was the cumulative live-birth rate. The latter was calculated as the number of women who achieved the first live birth (>28 weeks of gestation) in FET cycles or in subsequent FET cycles divided by the two groups of patients.

Other variables that we analyzed were the number of oocytes retrieved, the number of MII oocytes, as well as the rates of fertilization, implantation, clinical pregnancy, and miscarriage.

The “fertilization rate” was defined as the number of fertilized oocytes divided by the total number of the retrieved oocytes. The implantation rate was calculated by dividing the total number of fetal-cardiac-activity events detected by the total number of transferred embryos. “Clinical pregnancy” was defined as a pregnancy confirmed by ultrasound visualization of the gestational sac between the week-5 and−6 of gestation. The “miscarriage rate” was defined as the number of cases with pregnancy loss within 28 weeks of gestation starting from the day of oocyte fertilization divided by the number of clinical pregnancies. The “live-birth rate” was defined as the total number of the cases with at least one baby born after 28 weeks of gestation divided by the total number of FET cycles. The “cumulative live-birth rate” was calculated as the first live birth achieved after all cycles having an embryo transfer (fresh cycles as well as thawing cycles) among the two groups.

### Statistical Analyses

Data analyses were carried out using SPSS 21.0 (IBM, Armonk, NY, USA). Samples were assessed by the Shapiro–Wilk test to determine the normality of the distribution. Based on the results, parametric tests were preferred. Continuous variables are presented as the mean ± SD, and then compared using the Student's *t*-test or Mann–Whitney *U*-test. For categorical variables, the values are presented as frequencies and percentages. The chi-square test was used for comparisons between groups. *p* < 0.05 was considered significant.

## Results

A total of 469 women met the inclusion criteria and were included in the analysis (270 in the dual-trigger group and 199 in the hCG-alone group). The baseline characteristics and demographics did not differ significantly between the dual-trigger group and hCG-alone group in terms of age, BMI, or duration and cause of infertility (Table 1). In addition, there was no significant difference regarding the antral follicle count or FSH level on day-3 between the two groups.

**Table 1:** Comparison between the dual-trigger group and hCG-alone group: characteristics of patients at baseline.

	**Dual trigger**	**hCG alone**	***p***
	**(*n* = 270)**	**(*n* = 199)**	
Age (years)	32.66 ± 3.53	33.24 ± 3.64	0.08
BMI (kg/m^2^)	22.66 ± 2.69	22.72 ± 2.69	0.81
Duration of infertility (years)	3.79 ± 2.94	3.64 ± 2.97	0.61
Antral follicle count	10.60 ± 5.41	10.55 ± 4.50	0.91
FSH (IU/L) at day-3	7.17 ± 1.63	7.12 ± 1.38	0.83
Cause of infertility			0.06
Male factor	60 (22.20%)	39 (19.6%)	
Tubal factor	89 (33.00%)	55 (27.60%)	
Ovulation dysfunction	23 (8.50%)	33 (16.50%)	
Endometriosis	17 (6.30%)	16 (8.00%)	
Combined	59 (21.90%)	42 (21.10%)	
Unexplained	22 (8.10%)	14 (7.00%)	
Fertilization			0.27
IVF	138 (51.10%)	112 (56.30%)	
ICSI	132 (48.9%)	87 (43.70%)	

*Values are the mean ± standard deviation or percentage*.

*HCG, human chorionic gonadotrophin; BMI, body mass index; FSH, follicle-stimulating hormone; IVF, in vitro fertilization; ICSI, intracytoplasmic sperm injection*.

The characteristics of ovarian stimulation for each group are presented in Table 2. There was a significant difference in the starting dose of gonadotropins used in stimulation for the dual-trigger group and hCG group (189.95 ± 54.92 vs. 217.71 ± 54.43, *p* = 0.00). Differences regarding the total dose of gonadotrophin stimulation, total number of days of gonadotrophin stimulation, progesterone level, or endometrial thickness at the day of triggering were not found. In comparison with the hCG group, women who received a dual trigger had a slightly higher number of: retrieved oocytes (11.24 ± 4.76 vs. 10.24 ± 4.27, *p* = 0.02); two pronuclear (2PN) embryos (7.37 ± 3.69 vs. 6.62 ± 3.26, *p* = 0.02); embryos obtained (4.45 ± 2.41 vs. 4.03 ± 2.20, *p* = 0.04). There was no significant difference in the fertilization rate, number of MII oocytes retrieved, or the number of top-quality embryos between the two groups.

**Table 2:** Comparison between the dual-trigger group and hCG-alone group: characteristics of ovarian stimulation.

	**Dual trigger**	**hCG alone**	***p***
	**(*n* = 270)**	**(*n* = 199)**	
Initial dose of gonadotropin (IU)	189.95 ± 54.92	217.71 ± 54.43	0.00
Total dose of gonadotropins (IU)	2,100.41 ± 721.56	2,170.53 ± 622.28	0.27
Duration of stimulation (d)	9.57 ± 1.88	9.76 ± 1.82	0.27
Progesterone (ng/mL) on trigger day	1.23 ± 0.69	1.12 ± 0.92	0.18
Endometrial thickness (mm) on trigger day	9.02 ± 2.20	9.47 ± 2.71	0.06
No. of oocytes retrieved	11.24 ± 4.76	10.24 ± 4.27	0.02
No. of MII oocytes retrieved	8.37 ± 4.44	7.67 ± 3.69	0.07
Fertilization rate (%)	84.40 ± 17.71	83.44 ± 22.20	0.60
No. of 2PN embryos	7.37 ± 3.69	6.62 ± 3.26	0.02
No. of embryos available	4.45 ± 2.41	4.03 ± 2.20	0.04
No. of top-quality embryos	1.53 ± 1.53	1.31 ± 1.40	0.10

*Values are the mean ± standard deviation*.

*HCG, human chorionic gonadotropin; MII, metaphase II; 2PN, two-pronuclear*.

Ninety-three women underwent FET cycles in the dual-trigger group compared with 71 women in the hCG-trigger group. A slightly higher abortion rate was observed in the dual-trigger group, but this difference was not significant (17.95 vs. 10.34%, *p* = 0.38) (Table 3). The implantation rate, clinical-pregnancy rate, and live-birth rate were similar between the two groups with respect to FET cycles (Table 3). Further, we analyzed the cumulative pregnancy outcome of the two groups. There was no significant difference among the two groups regarding the implantation rate, abortion rate, or cumulative live-birth rate (Table 4). The abortion rate was slightly higher in the dual-trigger group (8.73 vs. 6.81%, *p* = 0.37) compared with that in the hCG-trigger group.

**Table 3:** Reproductive outcome of cycles of fresh-embryo transfer in the study groups.

	**Dual trigger**	**hCG alone**	***p***
	**(*n* = 93)**	**(*n* = 71)**	
Implantation rate (%)	28.83% (47/163)	28.00% (35/125)	0.88
Clinical-pregnancy rate per ET (%)	41.94% (39/93)	40.85% (29/71)	0.89
Abortion rate (%)	17.95% (7/39)	10.34% (3/29)	0.38
Live-birth rate per ET (%)	34.41% (32/93)	36.62% (26/71)	0.77

*ET, embryo transfer; hCG, human chorionic gonadotropin*.

**Table 4:** Cumulative reproductive outcomes in the study groups.

	**Dual trigger**	**hCG alone**	***p***
	**(*n* = 270)**	**(*n* = 199)**	
No. of embryos transferred per patient	2.60 ± 1.22	2.61 ± 1.15	0.97
No. of embryo transfer cycles per patient	1.44 ± 0.64	1.42 ± 0.63	0.75
Implantation rate (%)	36.66% (250/682)	35.81% (183/511)	0.76
Abortion rate (%)	8.73% (33/378)	6.81% (19/279)	0.37
Cumulative live-birth rate	54.07% (146/270)	59.30% (118/199)	0.26
Fresh-cycle live birth	32	26	
Frozen-cycle live birth	114	92	

*hCG, human chorionic gonadotropin*.

## Discussion

We investigated the effect of a dual trigger using a GnRHa and hCG for final oocyte maturation on the clinical reproductive outcome in normal responder women. We demonstrated that a dual trigger of a GnRHa and hCG slightly increased the numbers of oocytes retrieved, 2PN embryos, and embryos available for normal responders in GnRH-antagonist IVF–ICSI cycles. The abortion rate was higher in the dual-trigger group than that in the hCG-only group, but this difference was not significant. Therefore, the cumulative live-birth rate was comparable between the dual-trigger group and hCG-alone group.

The number of MII oocytes retrieved and the number of top-quality embryos were similar in both study groups. However, the number of oocytes retrieved, 2PN embryos, and embryos available was higher in the dual-trigger group. These data suggested that the dual trigger could improve the quantity of oocytes and embryos. Compared with hCG alone, administration of a GnRHa induces an increase in endogenous levels of LH and FSH that resembles the natural mid-cycle surge of gonadotropins. The surge in FSH level could activate resumption of oocyte meiosis and cumulus expansion at the final stage of oocyte maturation (15). In addition, a GnRHa trigger can activate the GnRH receptors on granulosa cells, which may regulate ovulation (16). Our results are consistent with data from several studies focusing on the use of a dual trigger for oocyte maturation in normal responders (1, 11, 12, 17). Several studies have shown no significant difference in the number of MII oocytes or the number of 2PN oocytes with use of a dual trigger in comparison with that using hCG alone. However, this disparity in results this may be due to the small sample sizes in such studies and the heterogeneity of the infertile population (18, 19).

We detailed the pregnancy outcome not only in FET cycles but also in FET cycles. In FET cycles, there was no significant difference between the dual-trigger group (93 patients) and hCG-alone group (71 patients) in terms of the rate of implantation, clinical pregnancy, or live births. The results indicated that in FET cycles, the dual trigger for oocyte maturation was not superior to a hCG trigger. Despite the slightly higher number of oocytes retrieved (11.24 vs. 10.24) and higher number of embryos available (4.45 vs. 4.03) in the dual-trigger group, these advantages did not result in a higher rate of implantation or live births. Furthermore, a much higher abortion rate was demonstrated in the dual-trigger group (17.95 vs. 10.34%), but this difference was not significant.

Our results are in accordance with those of Zhou et al. which reported no significant difference between the two study groups regarding the rates of implantation, clinical pregnancy, or live births in a retrospective cohort study involving 220 patients in a dual-trigger group and 110 control patients (12). In addition, randomized controlled trials (RCTs) by Shymaa et al. and Alleyassin et al. explored the effect of a dual trigger upon oocyte maturation and pregnancy outcome in FET cycles in normal responders (17, 20). A similar rate of implantation and clinical pregnancy was shown between the two groups in both RCTs, and a similar live-birth rate in FET cycles was demonstrated in the RCT by Shymaa and colleagues (which comprised 120 patients). Conversely, Lin and coworkers and Kim et al. reported that a dual-trigger group had significantly improved rates of implantation, clinical pregnancy, and live births (11, 21). Thus, in FET cycles, the benefits of a dual trigger for oocyte maturation in normal responders are controversial.

The cumulative birth rate can provide an all-inclusive success rate for ART, so we focused on the cumulative live-birth rate as the primary outcome. In the present study, all patients were followed up for 3 years. A total of 177 patients in the dual-trigger group and 128 patients in hCG-alone group received FET. The cumulative live-birth rate was calculated by including the first live birth generated during the complete IVF cycle (FET) as the numerator, and the denominator was defined as all women enrolled in the two groups. The cumulative live-birth rate was comparable between the two groups (54.07% in the dual-trigger group vs. 59.30% in the hCG-alone group, *p* = 0.26). Shymaa and colleagues explored the cumulative live-birth rate after FET cycles. A significant difference was not observed between the two groups regarding the cumulative pregnancy rate or cumulative live-birth rate in the RCT, in which only 19 patients (11 for the dual-trigger group vs. 8 in the hCG-alone group) received FET. Hence, a dual trigger with a GnRHa and hCG for oocyte maturation was not superior to a hCG-only trigger for normal responders in GnRH-antagonist cycles with respect to the cumulative pregnancy rate.

The strengths of our study were: (i) a large patient cohort; (ii) long follow-up; (iii) calculation of the cumulative live-birth rate in the two groups, which provided an all-inclusive success rate for ART. The main limitation of our study was its retrospective design and non-original. In addition, prospective RCTs with large study cohorts are needed to determine the exact impact of the trigger of final oocyte maturation in normal responders undergoing ART cycles.

## Conclusions

A dual trigger using a GnRHa and hCG can slightly improve the number of oocytes retrieved and the number of embryos for normal responders using GnRH-antagonist IVF cycles. However, a clinical benefit was not observed in terms of the cumulative live-birth rate.

## Data Availability Statement

The raw data supporting the conclusions of this article will be made available by the authors, without undue reservation.

## Ethics Statement

The studies involving human participants were reviewed and approved by the institutional review board of Peking University Peoples' Hospital. Written informed consent for participation was not required for this study in accordance with the national legislation and the institutional requirements.

## Author Contributions

HS and HH conceived and designed this study. FG and YW contributed to the acquisition and analyses of data. FG also contributed to helped to draft the manuscript. MF, QZ, and YR were responsible for data collection. HH contributed to revising the manuscript. All authors contributed to the article and approved the submitted version.

## Conflict of Interest

The authors declare that the research was conducted in the absence of any commercial or financial relationships that could be construed as a potential conflict of interest.

## References

[1] Schachter M Friedler S Ron-El R Zimmerman AL Strassburger D Bern O. Can pregnancy rate be improved in gonadotropin-releasing hormone (GnRH) antagonist cycles by administering GnRH agonist before oocyte retrieval? A prospective, randomized study. Fertil Steril. (2008) 90:1087–93. 10.1016/j.fertnstert.2007.07.1316 18023439

[2] Cerrillo M Pacheco A Rodríguez S Gómez R Delgado F Pellicer A. Effect of GnRH agonist and hCG treatment on VEGF, angiopoietin-2, and VE-cadherin: trying to explain the link to ovarian hyperstimulation syndrome. Fertil Steril. (2011) 95:2517–9. 10.1016/j.fertnstert.2010.12.054 21269615

[3] Gonen Y Balakier H Powell W Casper RF. Use of gonadotropin-releasing hormone agonist to trigger follicular maturation for in vitro fertilization. J Clin Endocrinol Metab. (1990) 71:918–22, 10.1210/jcem-71-4-918 2119392

[4] Itskovitz J Boldes R Levron J Erlik Y Kahana L Brandes JM. Induction of preovulatory luteinizing hormone surge and prevention of ovarian hyperstimulation syndrome by gonadotropin-releasing hormone agonist. Fertil. Steril. (1991) 56:213–20. 10.1016/S0015-0282(16)54474-4 1906406

[5] Kol S Humaidan P. GnRH agonist triggering: recent developments. Reprod Biomed Online. (2013) 26:226–30. 10.1016/j.rbmo.2012.11.002 23337420

[6] Kolibianakis EM Schultze-Mosgau A Schroer A Van Steirteghem A Devroey P Diedrich K. A lower ongoing pregnancy rate can be expected when GnRH agonist is used for triggering final oocyte maturation instead of HCG in patients undergoing IVF with GnRH antagonists. Human Reprod. (2005) 20:2887–92. 10.1093/humrep/dei150 15979994

[7] Humaidan P Ejdrup Bredkjær H Bungum L Bungum M Grøndahl ML Westergaard L. GnRH agonist (buserelin) or hCG for ovulation induction in GnRH antagonist IVF/ICSI cycles: a prospective randomized study. Hum Reprod. (2005) 20:1213–20. 10.1093/humrep/deh765 15760966

[8] Shapiro BS Daneshmand ST Garner FC Aguirre M Thomas S. Gonadotropin-releasing hormone agonist combined with a reduced dose of human chorionic gonadotropin for final oocyte maturation in fresh autologous cycles of in vitro fertilization. Fertil Steril. (2008) 90:231–3. 10.1016/j.fertnstert.2007.06.030 17981269

[9] Shapiro BS Daneshmand ST Garner FC Aguirre M Hudson C. Comparison of “triggers” using leuprolide acetate alone or in combination with low-dose human chorionic gonadotropin. Fertil Steril. (2011) 95:2715–7. 10.1016/j.fertnstert.2011.03.109 21550042

[10] Griffin D Benadiva C Kummer N Budinetz T Nulsen J Engmann L. Dual trigger of oocyte maturation with gonadotropin-releasing hormone agonist and low-dose human chorionic gonadotropin to optimize live birth rates in high responders. Fertil Steril. (2012) 97:1316–20. 10.1016/j.fertnstert.2012.03.015 22480822

[11] Lin MH Wu FS Lee RK Li SH Lin SY. Dual trigger with combination of gonadotropin-releasing hormone agonist and human chorionic gonadotropin significantly improves the live-birth rate for normal responders in GnRH-antagonist cycles. Fertil Steril. (2013) 100:1296–302. 10.1016/j.fertnstert.2013.07.1976 23993928

[12] Zhou X Guo P Chen X Ye D Liu Y Chen S. Comparison of dual trigger with combination GnRH agonist and hCG versus hCG alone trigger of oocyte maturation for normal ovarian responders. Int J Gynaecol Obstetr. (2018) 141:327–31. 10.1002/ijgo.12457 29388691

[13] Sükür YE Ulubaşoglu H Ilhan FC Berker B Sönmezer M Atabekoglu CS. Dual trigger in normally-responding assisted reproductive technology patients increases the number of top-quality embryos. Clin Exp Reprod Med. (2020) 47:300–35. 10.5653/cerm.2020.03804 33113599 PMC7711097

[14] Gardner DK Lane M Stevens J Schlenker T Schoolcraft WB. Blastocyst score affects implantation and pregnancy outcome: towards a single blastocyst transfer. Fertil Steril. (2000) 73:1155–8. 10.1016/S0015-0282(00)00518-5 10856474

[15] Kol S Humaidan P. LH (as HCG) and FSH surges for final oocyte maturation: sometimes it takes two to tango? Reprod Biomed Online. (2010) 21:590–2. 10.1016/j.rbmo.2010.06.031 20851052

[16] Maggi R Cariboni AM Marelli MM Moretti RM Andre V Marzagalli M. GnRH and GnRH receptors in the pathophysiology of the human female reproductive system. Hum Reprod Update. (2016) 22:358–81. 10.1093/humupd/dmv059 26715597

[17] Ali SS Elsenosy E Sayed GH Farghaly TA Youssef AA Badran E. Dual trigger using recombinant HCG and gonadotropin-releasing hormone agonist improve oocyte maturity and embryo grading for normal responders in GnRH antagonist cycles: randomized controlled trial. J Gynecol Obstetr Hum Reprod. (2020) 49:101728. 10.1016/j.jogoh.2020.101728 32173633

[18] Mahajan N Sharma S Arora PR Gupta S Rani K Naidu P. Evaluation of dual trigger with gonadotropin-releasing hormone agonist and human chorionic gonadotropin in improving oocyte maturity rates: a prospective randomized study. J Hum Reprod Sci. (2016) 9:101–6. 10.4103/0974-1208.183506 27382235 PMC4915279

[19] Decleer W Osmanagaoglu K Seynhave B Kolibianakis S Tarlatzis B Devroey P. Comparison of hCG triggering versus hCG in combination with a GnRH agonist: a prospective randomized controlled trial. Facts Views Vision Obstetr Gynnaecol. (2014) 6:203–9. 10.1186/1472-6874-12-27):27 25593695 PMC4286859

[20] Alleyassin A Ghasemi M Aghahosseini M Safdarian L Sarvi F Almasi-Hashiani A. Final oocyte maturation with a dual trigger compared to human chorionic gonadotropin trigger in antagonist co-treated cycles: a randomized clinical trial. Middle East Fertil Soc J. (2018) 23:199–204. 10.1016/j.mefs.2018.01.001

[21] Kim CH Ahn JW You RM Kim SH Chae HD Kang BM. Combined administration of gonadotropin-releasing hormone agonist with human chorionic gonadotropin for final oocyte maturation in GnRH antagonist cycles for in vitro fertilization. J Reprod Med. (2014) 59:63–8. 24597289

